# Prevalence of Epstein‐Barr Virus, Cytomegalovirus, and Periodontopathic Bacteria in Patients With Periodontitis: A Case–Control Study

**DOI:** 10.1002/cre2.70084

**Published:** 2025-02-06

**Authors:** Alicia Herrero‐Sánchez, Evelina Haroyan‐Darbinyan

**Affiliations:** ^1^ Private Practice Madrid Spain; ^2^ Faculty of Dentistry Complutense University of Madrid (U.C.M) Madrid Spain; ^3^ Faculty of Dentistry University Alfonso X el Sabio (U.A.X) Madrid Spain

**Keywords:** cytomegalovirus, Epstein‐Barr virus, herpesvirus, periodontitis

## Abstract

**Objectives:**

Opportunistic viruses such as cytomegalovirus (CMV) and Epstein‐Barr virus (EBV) have been detected in gingival crevicular fluid (GCF) and saliva of patients with periodontal disease. However, the relationship between herpesviruses and periodontitis remains obscure. The aim of this case–control study was the detection and association of CMV and EBV with periodontitis.

**Material and Methods:**

Forty‐eight adults were included in this study: 24 patients with periodontitis (CP) and 24 periodontally healthy individuals (HS). All patients underwent periodontal examination, including probing depth (PD), clinical attachment loss (CAL), plaque index (PI), and bleeding on probing (BOP). Subgingival biofilm samples were collected from all patients and real‐time PCR was performed for viral and bacterial detection. The odds ratio (OR) was calculated, and the chi‐squared test or Fisher's exact test was performed to analyze the significant differences.

**Results:**

EBV was detected only in one healthy patient meanwhile no CMV was found. No statistically significant differences were found between the periodontal clinical parameters of EBV‐positive patients and the negative ones: PI (*p* = 0.090), PD (*p* = 0.857), CAL (*p* = 0.801), and BOP (*p* = 0.104). Except for *Aggregatibacter actinomycetemcomitans (Aa), Porphyromonas gingivalis (Pg), Tannerella forsythia (Tf), Prevotella intermedia (Pi),* and *Treponema denticola (Td)* showed a statistically significant association (*p* < 0.001) with the clinical periodontal parameters. *Aa* presence was not statistically associated with periodontal sites (*p* < 0.296). *Tf and Pg* were the most frequently detected periodontopathic bacteria in the CP group (91.7% sites).

**Conclusion:**

The present case–control study showed that the prevalence of EBV and CMV did not show significant differences between the groups evaluated in the Spanish population.

## 1 Introduction

Periodontitis is a polymicrobial and chronic inflammatory disease that affects the periodontium and the supporting tissues of the teeth (Socransky and Haffajee 2005). This highly prevalent condition is caused primarily by pathogenic bacterial species located in the subgingival niche that elicit a complex inflammatory and immune response in the host (Kornman 2008). Consequently, periodontitis results in a deep crevice and gingival pocket formation (Pihlstrom, Michalowicz, and Johnson 2005). If untreated, the disease's progression leads to the destruction of supporting connective tissue and alveolar bone, which is a major cause of tooth loosening and tooth loss in adults (Helal et al. 2019). Moreover, in recent years, growing evidence shows that periodontitis can exacerbate systemic inflammatory diseases, such as atherosclerosis (Yang et al. 2018), diabetes mellitus (Chien et al. 2023), and chronic neurological disorders (Bai et al. 2023; Salhi et al. 2023) and even lead to adverse pregnancy outcomes (Wang et al. 2023; Figuero, Han, and Furuichi 2020).

The triggering of periodontal diseases is marked by an increase in the complexity of the microbiome (Ly et al. 2014; Costalonga and Herzberg 2014). Classically, periodontopathic bacteria, such as *Aggregatibacter actinomycetemcomitans (Aa), Porphyromonas gingivalis (Pg), Tannerella forsythia (Tf), Prevotella intermedia (Pi),* and *Treponema denticola (Td),* have been closely associated with the disease (Könönen and Müller 2014). However, periodontitis progresses by recurrent acute episodes followed by periods of remission (Socransky et al. 1984), suggesting that other pathogens than periodontopathic bacteria may be involved (Botero et al. 2020). Nonetheless, the presence of these microorganisms in subjects with no evidence of disease progression suggests that the disease is not the result of the sole presence of the bacteria but the net effect of the immune response and the inflammatory processes (Cekici et al. 2014).

In recent years, certain human herpesviruses (HHV), have been closely associated with the etiology and pathogenesis of a variety of periodontal diseases. Particularly, growing evidence supports that cytomegalovirus (CMV) and Epstein‐Barr virus (EBV) may cooperate with certain putative periodontopathic bacteria in the etiopathogenesis of severe types (Slots 2019; Chen, Feng, and Slots 2020; Saygun et al. 2008; Imai and Ogata 2020). In this regard, the above‐mentioned viruses have been found at a higher frequency in gingival crevicular fluid and saliva of periodontal patients when compared to healthy control subjects (Saygun et al. 2008; Contreras, Nowzari, and Slots 2000; Kato et al. 2013, 2015). Moreover, EBV and CMV displayed increased prevalence at deep periodontal pockets with periodontitis (Vincent‐Bugnas et al. 2013); thus, periodontal pockets may serve as a reservoir of viral organisms.

The exact pathophysiological mechanism implicated in the bacterial–viral interaction is unknown. On the one hand, herpesviruses are immunosuppressive; hence, they may alter host defense cells in the periodontium and lead to an overgrowth of periodontopathic bacteria (Chen, Feng, and Slots 2020; Contreras, Nowzari, and Slots 2000; Chalabi et al. 2010). In this regard, EBV seems to infect B lymphocytes and gingival epithelial cells (Imai and Ogata 2020; Kato et al. 2013), whereas CMV has been discovered in periodontal monocytes, macrophages, and T lymphocytes. Thus, EBV‐ and CMV‐infected periodontal sites show significant associations with the presence of periodontal pathogens (Slots and Contreras 2000). On the other hand, the reactivation of the above‐mentioned viruses in combination with other infectious agents may release pro‐inflammatory cytokines and chemokines, such as interleukin 1‐β, tumor necrosis factor α, and prostaglandins. Thus, these cytokines, due to their ability to cause inflammation and induce bone resorptions may ultimately lead to periodontal tissue destruction (Slots and Contreras 2000; Maulani et al. 2021).

Furthermore, the prevalence of different genotypes of CMV and EBV differs significantly by geography and ethnicity groups (Blazquez et al. 2021; Cannon, Schmid, and Hyde 2010; Wu et al. 2007). In fact, CMV seroprevalence was highest in South America, Africa, and Asia, and lowest in Western Europe (Cannon, Schmid, and Hyde 2010). Moreover, few studies have addressed the molecular identification of the herpesviruses after the updates of the new Classification of Periodontal and Peri‐Implant Diseases (Slots and Rams 2023; Hamada et al. 2023), and to the author's knowledge, none have compared CMV and EBV occurrence in patients with periodontitis in the Spanish population.

Therefore, the aim of the present study was to investigate the detection of CMV and EBV in healthy subjects (HS) and periodontal patients and to assess the association of the five periodontopathic bacteria to the target herpesviruses in the population of Madrid, Spain. Thus, the null hypothesis tested was that patients with generalized periodontitis and healthy control subjects showed no significant differences concerning the prevalence of EBV and CMV in subgingival plaque samples.

## 2 Materials and Methods

A case–control study was conducted in full accordance with the requirements of Helsinki Declarations (World Medical Association 2013). The study was approved by the Ethical Committee of Clinical Research, Health Research Institute of the Hospital San Carlos Madrid, Spain (Research Ethics Committee Approval Reference Number: CEIC 11.006.E). Written informed consent was obtained from all participants, once all procedures had been fully explained to the patients.

### 2.1 Sample Size Calculation

The sample size calculation to establish the required number of patients per group that would allow the detection of statistically significant differences was performed based on CMV virus prevalence of 45% in a cohort of periodontally healthy patients according to Kato et al (2013). A power of 80%, an expected odd ratio (OR) of 7.069 according to Maulani et al. (2021), and a significance level of *α* = 0.05 were considered. As a result, the minimum number of necessary subjects, with a case–control ratio of one, was *n* = 24 in each group.

### 2.2 Study Population

A total of 48 adults were recruited for this study from September 2016 to September 2017. Twenty‐four patients with Stage I and II generalized periodontitis (case group; CP) were consecutively included from those patients attending the Graduate Periodontal Clinic of Periodontology, Faculty of Dentistry, University Complutense of Madrid. The control group consisted of 24 periodontally healthy individuals (control group; HS) from the Reception Department, Faculty of Dentistry, University Complutense of Madrid.

### 2.3 Inclusion and Exclusion Criteria

The established inclusion criteria were the following: (a) patients between 20 and 70 years of age, (b) Caucasian ethnicity, (c) presence of more than 16 natural teeth excluding third molars, (d) patients with the diagnosis of Stage I and II periodontitis based on clinical and radiographic findings (CP group) (Yang et al. 2018) and periodontally HS.

Criteria for exclusion included (e) presence of systemic conditions, (f) patients who were pregnant or lactating, (g) subjects who used systemic antibiotic therapy within the previous 3 months, (h) patients with smoking habits, (i) presence of acute periodontal or dental pathologies, and (j) patients who received periodontal treatment in the last year.

### 2.4 Identification of Periodontal Cases and Healthy Controls

Periodontal examination included probing depth (PD) (Lindhe et al. 1982), clinical attachment level (CAL), bleeding on probing (BOP), and Plaque Index (O'Leary, Drake, and Naylor 1972). The PD and CAL were measured with a CPC‐12 probe (Hu‐Friedy, Chicago, IL, USA). All clinical measurements were performed on six sites per tooth, namely mesio‐buccal, mid‐buccal, disto‐buccal, mesio‐lingual, mid‐lingual, and disto‐lingual. Radiographic assessment of alveolar bone levels was obtained using 18‐radiograph full mouth series. Periodontal examination and subgingival sampling were performed by a single operator (A.H.S.).

The criteria for the diagnosis of the CP group were based on the 2018 Update to the 1999 Classification of Periodontal Diseases (Caton et al. 2018): patients with interdental CAL ≤ 4 mm at the site of greatest loss, a maximum PD ≤ 5 mm, and mostly horizontal bone loss with up to 33% of the coronal third. Periodontally HS were identified if BOP in less than 10% of sites were present with no PD ≤ 3 mm and the observed CAL must be ascribed to non‐periodontal causes.

### 2.5 Subgingival Sample Collection

Gingivo‐crevicular fluid (GCF) samples were consecutively collected from all subjects. Patients were asked to avoid food and drink ingestion 2 h before sample collection. The supragingival plaque was gently removed with sterile cotton pellets, then sample sites were isolated with cotton rolls, and dried before removing subgingival specimens (Kato et al. 2013). In the CP group, patients provided four subgingival samples, from the most accessible periodontal pockets with the deepest PD, one from each quadrant (Wu et al. 2007). In the HS group, samples were harvested from mesio‐buccal site of the first molars of each quadrant, and when absent, from the adjacent second molars. Two consecutive sterile endodontic paper points (Maillefer, Ballaigues, Switzerland) of size #30 were inserted into the depth of the pre‐selected sulcus/pocket and retained for 20 s. Harvested specimens were stored in a vial and stored at −80°C until processing (Konstantinidis et al. 2005).

### 2.6 Virological Analysis

A qualitative real‐time polymerase chain reaction (PCR) method was used to detect the DNA of CMV and EBV (*RealCycler* CMEB‐U, Progenie Molecular, Valencia, Spain) (Saygun et al. 2008). This viral analysis approach is a well‐established tool in microbiological diagnostic testing in periodontal treatments and related research (Konstantinidis et al. 2005; Kubar et al. 2005). The *RealCycler* CMEB‐U is an in vitro diagnostic kit that uses species‐specific primers and probes (Taqman probes) that are marked with fluorophores and a quencher dye. The Taqman probes emit fluorescence in the case of amplification. The system includes an internal control CHIC (Competitive Heterologous Internal Control) to prevent false negatives due to reaction inhibition. Each PCR experiment contained one negative control, which consisted of elution water. Furthermore, the diagnostic kit included the AmpliMix CMEB Positive Control, which consists of a DNA artificial construction of EBV and CMV. The amplification of EBV is detected with FAM fluorophore, CMV with TxR, and the internal control with HEX fluorophore.

The PCR amplification was performed in a DNA thermal cycler (Smart Cycler, Cepheid, Sunnyvale, CA, USA). Briefly, 6 μL of the extracted DNA sample or internal control was added to each reaction tube containing a ready‐to‐use amplicon sequencing mixture (AmpliMix, Progenie Molecular). The PCR conditions were as follows: an initial denaturation at 95°C for 15 min, followed by 45 cycles at 95°C for 15 s, at 60°C for 30 s, and at 72°C for 30 s. Sample results obtained were interpreted with GeneXpert Dx 4.6 (Cepheid, Sunnyvale, CA, USA) following the *RealCycler* CMEB‐U diagnostic kit instructions.

### 2.7 DNA Isolation

For the detection of five periodontopathic bacteria (*Aa, Pg, Tf, Pi*, and *Td*) and EBV and CMV pathogens, the isolation of DNA is necessary. To achieve this goal, the previously harvested specimens were thawed and transferred to a microcentrifuge tube containing 200 μL saline solution, 200 μL Proteinase‐K, and 10 μL lysis buffer (AL buffer, Qiagen). DNA extraction from subgingival samples was carried out using the QIAamp DNA Mini Kit (Qiagen, Venlo, Netherlands) following the manufacturer's instructions. Subsequently, the samples were stored at −20°C until further processing.

### 2.8 Bacterial Detection

Multiplex PCR followed by reverse hybridization was performed to identify the five periodontopathic bacteria (Urbán et al. 2010). The micro‐IDent (Hain Lifescience, Nehren, Germany) commercial test systems were used for the qualitative and semiquantitative detections of *Aa, Pg, Tf, Pi*, and *Td* according to the manufacturer's guidelines and following well‐established bacterial detection protocols (Urbán et al. 2010; Eick et al. 2011). The PCR cycler Trio Thermoblock (Biometra, Goettingen, Germany) was used for DNA amplification. Consecutive reverse hybridization based on DNA‐STRIP technology was performed according to the micro‐IDent protocol (Hain Lifescience). Hybridization products were visualized by adding substrate for alkaline phosphatase containing dimethyl‐sulfoxide (Eick et al. 2011). The color reactions produced as a result were registered. According to the manufacturer, the threshold for the bacterium detection test is set to 10^3^ for *Aa* and 10^4^ for *Pg, Tf, Tg,* and *Pi*. The interpretation of the results was performed by using an evaluation sheet supplied by the kit. The DNA‐based band analysis and microbial identification procedures were performed by two independent and blinded experts.

### 2.9 Statistical Analysis

Data were collected and analyzed using the statistical software Stata 16 (Stata Corp, College Station, USA). The unit of analysis was the patient/subject. Descriptive statistics were reported as means with standard deviations (SD) and the frequencies were expressed as percentages. As the Shapiro−Wilk test confirmed the normality of distributions, parametric probes were selected (Vetter 2017). Levene's test was performed to assess the equality of variances (Soave and Sun 2017). Demographic and clinical parameters were compared between the CP and HS groups using a chi‐square test or Fisher's exact test for categorical responses and an independent sample *t*‐test quantitative data. Qualitative results as the presence or absence of CMV, EBV, and the periodontopathic bacteria in both groups (HS and CP) were presented with contingency tables. The odds ratio (OR) was calculated, and the chi‐squared test and Fisher's exact test were performed to determine the association between the viral detection frequency in both study groups. The student's *t*‐test and the Mann−Whitney test were used to compare the differences between periodontal parameters, such as PI, PD, CAL, and BOP, in patients with or without the presence of periodontopathic bacteria. The significance level was set in advance at *α* = 0.05 (Kubar et al. 2005).

## 3 Results

From a total of 492 patients screened between September 2018 to May 2019, 48 participants were included in this study: 24 patients with Stage I and II periodontitis (CP group) and 24 periodontally healthy individuals (HS group). Table 1 describes the demographic characteristics of the study subjects. The patient's age ranged from 28 to 69 in the CP group and from 27 to 69 in the HS group. Patients in the CP group were significantly older (*p* < 0.001) than subjects in the HS group. Table 2 shows the mean clinical parameters of the whole mouth and those of harvested sites. Periodontitis patients (CP group) failed to demonstrate any CMV presence. Only one patient revealed EBV counts in the HS group. The frequency of detection of EBV, CMV, and periodontopathic bacteria is displayed in Table 3. The differences between clinical factors in the EBV‐positive and ‐negative groups are exposed in Table 4. The clinical periodontal and microbiological parameters of the study population are shown in Table 5. A statistically significant association was observed between the presence of *Pg, Tf, Pi,* and *Td* and the clinical periodontal parameters, respectively (PI, PD, CAL, and BOP) (*p* < 0.001). Whereas patients with the presence of *Aa* did not display differences in PI (*p* = 0.545), PD (*p* = 0.856), CAL (*p* = 0.376), and BOP (*p* = 0.243; Table 5). The only EBV‐positive subject was periodontally healthy. The mean PI was 3.57%, mean PD was 2.13%, mean CAL was 2.13 mm, and mean BOP was 0.59%.

**Table 1:** Patient‐based demographics of the study population.

	CP	HS	*p* value
*N* patients	24	24	
Patient age, mean (SD)	51.3 (9.8)	40.9 (8.7)	< 0.001^a^
Sex			
Females, *n* (%)	15 (62.5%)	12 (50.0%)	0.383^b^
Males, *n* (%)	9 (37.5%)	12 (50.0%)	
Remaining teeth, mean (SD)	25.3 (2.6)	27.5 (0.9)	< 0.001^a^

Abbreviations: CP, periodontitis cases group; HS, healthy subjects’ group.

^a^ Student's *t*‐test, equal variances assumed.

^b^ Pearson chi‐Square.

**Table 2:** Clinical parameters of the study subjects in both groups.

		Whole mouth examination (mean of all teeth)			Sample harvesting teeth (mean of 6 sites per tooth)		
Group		CP (*n* = 24)	HS (*n* = 24)	*p* value	CP	HS	*p* value
PI (%)		66.8 (16.4)	10.4 (4.8)	0.001^a^	98.0 (0.1)	0.0 (0.1)	< 0.001^a^
PD (mm)		3.5 (0.6)	1.9 (0.2)	< 0.001^a^	5.9 (1.1)	2.3 (0.6)	< 0.001^a^
CAL (mm)		4.1 (0.9)	2.0 (0.2)	< 0.001^a^	0.5 (0.7)	0.0 (0.0)	< 0.001^a^
BOP (%)		41.4 (15.0)	3.6 (2.5)	< 0.001^a^	91.0 (0.2)	0.0 (0.1)	< 0.001^a^

Abbreviations: BOP, bleeding on probe; CAL, clinical attachment level; PD, probing depth; PI, plaque index.

^a^ Student's *t*‐test, equal variances assumed.

**Table 3:** Frequency of detection of EBV, CMV, and periodontopathic bacteria in both groups.

Bacteria and viruses	CP *n* (%)	HS *n* (%)	*p* value
*Aa*	3 (12.5)	1 (4.2)	0.296^a^
*Pg*	22 (91.7)	3 (12.5)	< 0.001^a^
*Pi*	17 (70.8)	1 (4.2)	< 0.001^b^
*Tf*	22 (91.7)	9 (37.5)	< 0.001^a^
*Td*	19 (79.2)	1 (4.2)	< 0.001^b^
EBV	0	1 (4.2)	0.500^b^
CMV	0 (0.0)	0 (0.0)	1.000^b^

^a^ Pearson chi‐square.

^b^ Fisher's exact test.

**Table 4:** Clinical parameter differences in the EBV‐positive and ‐negative groups.

EBV presence	PI (%)	PD (mm)	CAL (mm)	BOP (%)
EBV (+)^a^	3.7 (1.0)	2.1 (0.7)	2.1 (0.8)	0.59 (0.1)
EBV (−)^b^	39.4 (30.8%)	2.7 (0.9)	3.0 (1.2)	23.9 (21.9)
*p* value^c^	0.090	0.857	0.801	0.104

Abbreviations: BOP, bleeding on probing; CAL, clinical attachment level; PD, probing depth; PI, plaque index

^a^ Mean of 6 sites per tooth (*n* = 1).

^b^ Mean of 6 sites per tooth (*n* = 47).

^c^ Student's *t*‐test, equal variances assumed.

**Table 5:** Comparison of clinical characteristics of the study population (*n* = 48) associated with the presence or absence of the five target periodontopathogens, respectively.

Bacterial presence	PI (%)	PD (mm)	CAL (mm)	BOP (%)
*Aa* (+) (*n* = 4)	47.7 (25.1)	2.8 (0.8)	3.6 (1.5)	34.83 (23.6)
*Aa* (−) (*n* = 44)	37.8 (31.50)	2.7 (0.9)	3.0 (1.2)	21.4 (21.6)
*p* value^a^	0.545^a^	0.856	0.376	0.243
*Pg* (+) (*n* = 25)	59.5 (23.8)	3.3 (0.7)	3.8 (1.1)	36.7 (19.1)
*Pg* (−) (*n* = 23)	15.9 (19.8)	2.1 (0.6)	2.1 (0.7)	7.1 (12.4)
*p* value^a^	< 0.001	< 0.001	< 0.001	< 0.001
*Pi* (+) (*n* = 18)	62.0 (19.6)	3.6 (0.7)	4.1 (1.0)	38.3 (23.6)
*Pi* (−) (*n* = 30)	24.6 (27.9)	2.2 (0.6)	2.4 (0.9)	13.0 (15.5)
*p* value^a^	< 0.001	< 0.001	< 0.001	< 0.001
*Tf* (+) (*n* = 4)	51.2 (28.8)	3.1 (0.9)	3.5 (1.2)	30.4 (21.1)
*Tf* (−) *(n* = 44)	15.7 (19.6)	2.0 (0.5)	2.2 (0.9)	8.1 (14.9)
*p* value^a^	< 0.001	< 0.001	< 0.001	< 0.001
*Td* (+) (*n* = 20)	63.4 (20.6)	3.5 (0.7)	4.0 (1.0)	41.2 (16.7)
*Td* (−) (*n* = 28)	19.5 (31.4)	2.2 (0.6)	2.3 (0.9)	9.2 (13.8)
*p* value^a^	< 0.001	< 0.001	< 0.001	< 0.001

Abbreviations: BOP, bleeding on probing; CAL, clinical attachment level; PD, probing depth; PI, plaque index.

^a^ Student's *t*‐test, equal variances assumed.

## 4 Discussion

Notwithstanding its chronic, polymicrobial, and host‐mediated nature, periodontitis is paradoxically manifested by alternating periods of exacerbation and remission (Emecen‐Huja et al. 2020; Hausmann and Jeffcoat 1988). Moreover, conventional periodontal therapies can be unpredictable and insufficient in the management of periodontal diseases, especially at sites with little plaque formation and rapid periodontal destruction (Maulani et al. 2021). Taken together, recent reports suggest that the complex periodontal microbiota may include pathogens other than the periodontopathic bacteria (Emecen‐Huja et al. 2020). In this sense, herpesviruses such as EBV and CMV have been closely associated with the etiopathogenesis of periodontitis (Chen, Feng, and Slots 2020; Slots, Kamma, and Sugar 2003). In fact, increased bacterial loads, especially *Pg* and *Tf*, have been observed in correlation with the presence of the above‐mentioned herpesviruses (Saygun et al. 2008; Slots, Kamma, and Sugar 2003). Seemingly, these viruses are associated with the severity of the disease (Saygun et al. 2008), since they are more likely to appear in patients with a higher BOP score (Idesawa et al. 2004), as well as in pocket depth, attachment loss, plaque and gingival index (Saygun et al. 2008).

However, research on the association between the viral presence and periodontal health and the periodontopathic bacteria in the Spanish population is scarce, especially after the implementation of the new classification of periodontal diseases. The present study aimed to assess the role of specific herpesvirus in the etiology of periodontitis. Our null hypothesis was accepted, as the prevalence of EBV and CMV did not display significant differences in the tested groups.

In this study, no sample site was CMV positive regardless of periodontal health (Table 3). Our findings are consistent with the very low detection rate of CMV (Watanabe et al. 2007; Dawson et al. 2009) as well as the infrequent presence of EBV in periodontitis sites (Dawson et al. 2009; Sunde et al. 2008). Particularly, a recent longitudinal study that followed a cohort of patients over a period of 6 months reported that only 4 of 1454 sample sites displayed CMV presence (0.3%), as well as EBV loads were low, being similar at disease sites that progressed compared to those that did not progress (Emecen‐Huja et al. 2020).

Similar to other studies, we did not find an association between plaque control scores and periodontal pocket depth in CMV‐positive patients, regardless of the higher prevalence of this herpesvirus observed in periodontal patients (Nakamura et al. 2020). Whereas several studies have reported a high prevalence of EBV (Chen, Feng, and Slots 2020; Saygun et al. 2008; Imai and Ogata 2020; Kato et al. 2013) and CMV (Imai and Ogata 2020; Mahendra et al. 2023), associating with an increased risk of periodontitis. Although the potential herpesviral‐bacterial coinfection has not been completely elucidated, their synergistic interaction is becoming increasingly supported by a body of evidence.

Particularly, herpesviruses seem to be markedly associated with periodontal clinical variables, such as CAL and increased pocket depth and bacterial counts, (Saygun et al. 2008; Kubar et al. 2005) together with the occurrence of major periodontopathic bacteria (Contreras et al. 1999).

However, the precise mechanisms of the viral contribution to the etiopathogenesis of periodontitis remain unraveled. Similar to periodontopathic bacterial infections, EBV has been suggested to stimulate the production of inflammatory cytokines in gingival fibroblast‐inducing osteoclast differentiation by contacting oral cells (Yokoe et al. 2022). Furthermore, the above‐mentioned viruses are thought to impair the immunoregulation of host cells, decreasing periodontal defenses and facilitating the colonization of periodontopathic bacteria (Muzammil et al. 2017). Hence, viral reactivation may downregulate immune responses, which together with the microbial overgrowth, might explain the cyclical pattern of periodontal breakdown, with periods of remission and exacerbation (Watanabe et al. 2007). In this line, Imai and Ogata (2020) suggest that butyric acid (BA) producing periodontopathic bacteria may be the key step in the EBV reactivation, as BA seems to regulate the switch between the latent and lytic infection forms of EBV. This etiological model assumes, therefore, that the intricate interplay of BA‐producing periodontopathic bacteria and EBV leads to the above‐described production of inflammatory cytokines, which collectively advances chronic periodontitis.

The inconsistencies regarding the prevalence of CMV and EBV in periodontal pockets could have diverse reasons, including study methodology, case definition of periodontitis, different geographical distribution, different methods for viral DNA detection, and sensitivity of DNA‐isolation techniques, as well as sampling techniques.

In our study, patients with periodontitis Stage I and II were included following the most recent classification (Caton et al. 2018), which does not discriminate between aggressive periodontitis and chronic periodontitis. However, most studies find a closer connection of aggressive periodontitis to the herpesvirus than to chronic periodontitis (Maulani et al. 2021; Li et al. 2017). Furthermore, the eligibility criteria to determine the case and control groups differed in many studies. In our study, clinical parameters (CAL, BOP, PD, and PI) were assessed to discriminate between the two study groups (Table 2). Moreover, four of five major periodontopathic bacteria (*Pg, Td, Tf,* and *Pi*) were strongly associated with the clinical periodontal parameters in this study population (Table 5). The variability in the clinical parameters considered to define a periodontitis case and the selection criteria among the different studies could have masked the severity of the disease. Some studies required at least 20 teeth to be included in the study, meanwhile others only required 10 or 9 teeth, and many others did not mention such a requirement (Maulani et al. 2021).

It should be noted that an important reason for discrepancies in the prevalence of herpesvirus is the disparity in the sampling (pooled sample/single site), the subgingival sample collection method (GCF or subgingival plaque), and sensitivity of the viral detection techniques, which could lead to a biased interpretation of the results (Emecen‐Huja et al. 2020). Indeed, most studies employed nested PCR (Kato et al. 2013; Saygun et al. 2002), which has been demonstrated to overestimate the viral copies at individual sites (Botero et al. 2020).

Disregarding methodological differences, our study may present limitations. First, the relatively small sample size could have reduced the representativeness of the population. Second, periodontitis Stage I and II cases may not reflect the broad spectrum of periodontal diseases, underestimating the scope of the viral–bacterial interaction. Another limitation is the fact that qualitative PCR was employed to investigate the presence or absence of herpesviruses, which could have affected the detection rate. Future research using virome sequencing analysis, and a larger sample size with a broader range of periodontitis cases could yield a better understanding of the association of the EBV and CMV and periodontal disease.

## 5 Conclusions

Within the limitations of this case–control study, the following conclusions may be drawn:

1. No significant association was found between EBV and CMV presence and periodontal health status in this specific population.
2. Nevertheless, the very low detection rate of EBV and CMV may be explained by the presence of patients with mild periodontitis and the sample collection method.
3. The target periodontal pathogens (*Pg, Tf, Pi,* and *Td)* showed the statistically strongest association with the clinical parameters of periodontitis.

## Author Contributions

A.H.S. performed the study design, methodology, data collection, data interpretation, review, and editing. E.H.D. performed the statistics and wrote the main manuscript.

## Ethics Statement

This study was approved by the Institutional Review Board of Hospital Clínico San Carlos Ethics Committee in Madrid (Ethics Committee Approval Reference Number: CEIC 11.006.E).

## Consent

Written informed consent was obtained from all patients.

## Conflicts of Interest

The authors declare no conflicts of interest.

## Data Availability

Data supporting the funding of this study are available upon request.
